# Positive Surgical Margin, HPV Persistence, and Expression of Both TPX2 and PD-L1 Are Associated with Persistence/Recurrence of Cervical Intraepithelial Neoplasia after Cervical Conization

**DOI:** 10.1371/journal.pone.0142868

**Published:** 2015-12-01

**Authors:** Hui Zhang, Tingguo Zhang, Zongbing You, Youzhong Zhang

**Affiliations:** 1 Department of Obstetrics and Gynecology, Qilu Hospital of Shandong University, Jinan, Shandong, China; 2 Department of Obstetrics and Gynecology, Affiliated Hospital of Taishan Medical College, Taian, Shandong, China; 3 Department of Pathology, Qilu Hospital of Shandong University, Jinan, Shandong, China; 4 Department of Structural & Cellular Biology, Tulane University, New Orleans, Louisiana, United States of America; 5 Department of Orthopaedic Surgery, Tulane University, New Orleans, Louisiana, United States of America; 6 Tulane Cancer Center and Louisiana Cancer Research Consortium, Tulane University, New Orleans, Louisiana, United States of America; 7 Tulane Center for Stem Cell Research and Regenerative Medicine, Tulane University, New Orleans, Louisiana, United States of America; 8 Tulane Center for Aging, Tulane University, New Orleans, Louisiana, United States of America; Georgetown University, UNITED STATES

## Abstract

**Objective:**

To determine the clinicopathologic and immunohistochemical predictors of the persistence/recurrence of cervical intraepithelial neoplasia (CIN) after cervical conization.

**Methods:**

Medical records of 502 patients who received cervical conization treatment of CIN between 2005 and 2012 were reviewed. The clinicopathologic parameters were analyzed using Cox hazard regression. Fifty patients with CIN persistence/recurrence were matched to 50 cases without CIN persistence/recurrence. These 100 cervical specimens were assessed for expression of insulin-like growth factor II messenger RNA (mRNA)-binding protein 3 (IMP3), targeting protein for xenopus kinesin-like protein 2 (TPX2), and programmed cell death-1 ligand-1 (PD-L1) using immunohistochemical staining.

**Results:**

Multivariate analysis found that the independent predictors of CIN persistence/recurrence were positive surgical margin (hazard ratio 5.777, 95% confidence interval 2.334–14.301, *p* < 0.001) and human papilloma virus persistence for 6 months (hazard ratio 20.685, 95% confidence interval 7.350–57.657, *p* < 0.001). Co-expression of TPX2 and PD-L1 was significantly higher in CIN persistence/recurrence group than the group without CIN persistence/recurrence (*p* = 0.013). The depth of glandular involvement (GI) was less than 3mm in about 86.8% (59/68) CIN2-3 lesions, However, No statistically significant associations between GI and persistence/recurrence were observed (P = 0.58).

**Conclusion:**

Positive surgical margin, HPV persistence, and expression of both TPX2 and PD-L1 are associated with persistence/recurrence of cervical intraepithelial neoplasia after cervical conization.

## Introduction

Cervical conization is now a widespread method for diagnosing and treating cervical intraepithelial neoplasia (CIN). Cold knife conization (CKC) and loop electrosurgical excision procedure (LEEP) are the two most common available techniques. Although cervical conization is thought to be extremely effective, 5% to 25% of the cases are left with persistent or recurrent high-grade lesions [1–4]. The risk of developing cervical cancer after treatment of CIN is five times higher than that in the general population [5]. Previous studies have suggested that the increased risk of cervical cancer may be associated with incomplete follow-up [1,6]. About half of the treated patients fail to comply with the follow-up schedules in the first two years [7], which poses a challenge in the post-conization surveillance protocol.

It has been suggested that positive margins, persistent human papilloma virus (HPV) infection, CIN grade, treatment type, glandular involvement, age and immunosuppression are the predictors for persistence/recurrence [1,8–12]. However, it is still difficult for the gynecologists to predict the outcome of each individual patient. Therefore, additional studies of molecular biomarkers besides the pathologic or demographic parameters may be of value for improving the outcome prediction.

A previous study showed that expression of insulin-like growth factor II messenger RNA (mRNA)-binding protein 3 (IMP3) is associated with poor prognosis in cervical cancer [13]. Targeting protein for xenopus kinesin-like protein 2 (TPX2) has been demonstrated to be overexpressed in cervical cancer [14]. Since both IMP3 and TPX2 have been associated with growth of cervical cancer, we speculated that they might also be associated with growth of CIN. A recent meta-analysis indicated that expression of programmed cell death-1 ligand-1 (PD-L1, also called B7-H1) is associated with poor prognosis of many cancers [15]. PD-L1 acts on programmed cell death-1 (PD-1) to inhibit activated T cells, thus suppressing immune function. We speculated that high PD-L1 expression might favor HPV infection and growth of abnormal cervical epithelial cells, hence being associated with persistence/recurrence of CIN after cervical conization. Therefore, we assessed the expression of IMP3, TPX2, and PD-L1 using immunohistochemistry (IHC) and determined their potential value as molecular biomarkers for the persistent or recurrent CIN lesions after conization. We hypothesized that IMP3 and TPX2 (via mechanisms of driving cervical epithelial cells growth) and PD-L1 (via mechanisms of promoting HPV infection and abnormal cervical epithelial cells growth) might be associated with the risks of persistent/recurrent CIN. We also evaluated whether any clinical or histopathologic variables were able to identify the subclass of patients with a high risk of persistence / recurrence.

## Materials and Methods

### Human Patient Data

We included all patients who underwent a CKC or LEEP procedure because of CIN at the Qilu Hospital of Shandong University from July 2005 to December 2012. Records were obtained retrospectively from the colposcopy computer database and the hospital’s patient database including information about patient’s age, parity, menopausal status, cone depth, punch biopsy histological grade, conization histological grade and margin status. All data were retrieved between February and May 2015. Using the name, admission number or the registration number of LEEP, we can identify the patients’ information in the archives. This study was approved by the Human Ethics Committee of the Qilu Hospital of Shandong University. A written informed consent regarding use of specimens for future research was obtained from all the patients.

Colposcopy was performed prior to conization. The largest diameter of the loop was chosen to be 25 mm and it was ensured that the excision of the epithelium was about 3–5 mm beyond the borderline of the lesion. The specimens were examined for cone depth, histologic grade, orientation and surgical margins state. Margins were reported as positive if any grade of CIN existed at or near (≤1 mm) the resection surface.

Post-conization follow-up included cervical Pap smear and Hybrid Capture II HPV DNA testing after 3, 6, and 12 months in the first-year, and then every 6–12 months. All women with abnormal cytology or/and consistent HPV infection for 1 year underwent a colposcopy-directed biopsy (CDB) and/or an endocervical curettage (ECC) if indicated. Any grade of CIN on the histological diagnosis or malignant transformation was considered as persistent/recurrent CIN lesion.

Some patients were excluded from this study based on the following exclusion criteria: 1) incompliance with regular post-conization follow-up (regular follow-up for at least 1 year); 2) cervical biopsy diagnosis attained from other hospitals without consultation; 3) specimens obtained from other than conization (e.g., hysterectomy); 4) micro-invasive cervical cancer; and, 5) glandular abnormalities. Totally 502 patients were included, from which 50 patients were found having CIN recurrence/persistence and another 50 patients without CIN persistence/recurrence were identified to match them. Thus, a total of 100 paraffin-embedded conization specimens were obtained from the Department of Pathology at the Qilu Hospital of Shandong University. Initial histopathological diagnosis on the H&E sections was re-reviewed by 2 gynecologic pathologists. The number of quadrants involvement and depth of gland involvement (GI) were also assessed. The depth of gland involvement (GI) was measured by the distance between the basement membrane of the surface epithelium and the deepest site of gland involvement (GI).

### Immunohistochemical (IHC) Staining

IHC staining was performed using the following antibodies from Abcam (Cambridge, MA, USA): mouse anti-TPX2 (Ab32795, 1:300 dilution), rabbit anti-IMP3 (Ab109521, 1:100 dilution), and mouse anti-PDL1 (Ab58810, 1:40 dilution). IHC was carried out with the avidin-biotin complex (ABC) using SP Kit (Vector Laboratories, Burlingame, CA, USA) according to the manufacturer’s instructions. Briefly, the reviewed sample was then submitted to consecutive cuts at 4 μm. Slides were dried at 60°C for 1 hour, de-paraffinized in xylene, re-hydrated through a graded ethanol series. Antigen retrieval was performed by boiling the tissues in 0.01M EDTA buffer at 95°C for 5 min. After blocking endogenous peroxidase activity, the slides were incubated with primary antibody overnight at 4°C refrigerator. Subsequently, secondary antibody was applied for 1 hour. Immunoreactive complexed were detected using diaminobenzidine chromogen. Finally, slides were counterstained with hemotoxylin, dehydrated in graded ethanol, cleared with xylene and mounted. The negative controls used the non-immune isotype serum instead of the primary antibodies, and the positive controls were the tissue sections that previously stained positive for the marker proteins. The IHC staining was evaluated based on the Allred scoring system [16]. The proportion scores were: 0, none; 1, less than one-hundredth; 2, one-hundredth to one-tenth; 3, one-tenth to one-third; 4, one-third to two-third; and 5, greater than two thirds. The intensity scores represented the estimated average staining intensity of positive tumor cells: 1, weak; 2, intermediate; and 3, strong. Ten high-power fields (x400 magnification) of each specimen were evaluated. The average intensity scores and proportion scores were added as the total Allred scores (range = 0 to 8).

### Statistical Analysis

Statistical analysis was performed using SPSS version 20.0 for Windows (SPSS Inc., Chicago, IL. USA). Discrete variables were expressed as median (range), categorical variables as number (percentage).Analysis of quantitative data was undertaken using Mann-Whitney U test. Analysis of categorical data was undertaken using Chi-square test or Monto Carlo exact test. Hazard ratio (HR) with 95% confidence interval (CI) was calculated. Multivariate analysis was achieved using Cox hazard regression. The Kaplan-Meier method was used to estimate the time to CIN persistence/recurrence. The log-rank test was used to test for possible differences between different groups. All *p* values were 2-tailed and *p* ≤ 0.05 was considered statistically significant.

## Results

A total of 502 patients were included in this study. The patients ranged from 21 to 71 years of age, with a mean age of 38.5 years. After a median follow-up of 33 months (range: 1–94 months), persistent/recurrent lesions were found in 50 patients (approximately 10%).One patient with CIN2 in LEEP specimen showed invasive cancer during follow-up one month later after LEEP. In order to better analyze variables potentially involved with CIN persistence/recurrence, the patients were divided into two groups according to their follow-up results (see Table 1). There were no significant variations in terms of age, parity, menopausal status, types of conization, punch biopsy, HPV infection, colposcopy, and histological results in conization specimens. On the other hand, patients who had a persistent/recurrent lesions had a higher rate of positive surgical margins (*p*<0.001) and a cone depth less than 18 mm (*p* = 0.049).

**Table 1:** Correlation between demography and histopathology with recurrence. CKC, cold knife conization; LEEP, loop electrosurgical excision procedure; CIN, cervical intraepithelial neoplasia; HPV, human papilloma virus.

Variable		No recurrence (n = 452)	Recurrence (n = 50)	P value
**Age (median and range)**		**39 (21–71)**	**38 (21–62)**	**0.844** ^**a**^
**Menopause**				**0.783** ^**b**^
	**Yes**	**23 (5.1%)**	**3 (6%)**	
	**No**	**429 (94.9%)**	**47 (94%)**	
**Parity (median and range)**		**1 (0–3)**	**1 (0–3)**	**>0.99** ^**a**^
**Conization method**				**0.454** ^**b**^
	**LEEP**	**148 (32.7%)**	**19 (38%)**	
	**CKC**	**304 (67.3%)**	**31 (62%)**	
**Cone depth (mm)**				**0.049** ^**b**^
	**≤18**	**162 (35.8%)**	**25 (50%)**	
	**>18**	**290 (64.2%)**	**25 (50%)**	
**Punch biopsy**				**0.325** ^**b**^
	**CIN1**	**29 (6.4%)**	**3 (6%)**	
	**CIN2**	**154 (34.1%)**	**12 (24%)**	
	**CIN3**	**269 (59.5%)**	**35 (70%)**	
**High risk HPV**				**0.289** ^**b**^
	**Yes**	**412(91.2%)**	**48 (96%)**		
	**No**	**40 (8.8%)**	**2(4%)**	
**Colposcopy adequacy**				**0.227** ^**b**^
	**Yes**	**354 (78.3%)**	**35 (70%)**	
	**No**	**98 (21.7%)**	**15 (30%)**	
**Cone pathology**				**0.271** ^**b**^
	**Normal**	**54 (11.9%)**	**3 (6%)**	
	**CIN1**	**67(14.8%)**	**6 (12%)**	
	**CIN2**	**105(23.2%)**	**9 (18%)**	
	**CIN3**	**226 (50%)**	**32 (64%)**	
**Surgical margins**				**<0.001** ^**b**^
	**Negative**	**402 (97.1%)**	**38 (82.6%)**	
	**Positive**	**12 (2.9%)**	**8 (17.4%)**	
**Follow-up (months, median, range)**		**34 (12–94)**	**12 (1–55)**	

^**a**^ **Pearson Chi-square test.**

^**b**^ **Mann-Whitney U test.**

In order to evaluate which variables could be considered as independent predictors for long-term recurrence after conization, we used the Cox proportional hazard model. We found that the independent predictors of CIN persistence/recurrence were positive surgical margin (HR 5.777, 95% CI 2.334–14.301, *p* < 0.001; Wald 14.386) and HPV persistence more than 6 months (HR 20.685, 95% CI 7.350–57.657, p < 0.001; Wald 33.131) (see Table 2).

**Table 2:** Multivariate analysis of factors for prediction of persistence/recurrence after CKC/LEEP. LEEP, loop electrosurgical excision procedure; CIN, cervical intraepithelial neoplasia; HPV, human papilloma virus.

		Multivariate analysis	
Variables	Wald	HR (95% CI)	*P* value ^a^
**Age (>35 years)**	**0.145**	**0.866 (0.414–1.811)**	**0.703**
**Vaginal delivery (≥1)**	**0.143**	**0.816 (0.285–2.335)**	**0.705**
**Menopause**	**0.359**	**1.454 (0.428–4.941)**	**0.549**
**Conization method (LEEP)**	**0.069**	**1.105 (0.523–2.335)**	**0.793**
**Cone pathology (CIN3)**	**0.773**	**1.197 (0.801–1.788)**	**0.379**
**Cone depth (≤18 mm)**	**0.607**	**0.756 (0.374–1.528)**	**0.436**
**Glandular involvement**	**0.096**	**1.140 (0.496–2.621)**	**0.757**
**Surgical margins (positive)**	**14.386**	**5.777 (2.334–14.301)**	**<0.001**
**HPV persistence (≥6 months)**	**33.131**	**20.586 (7.350–57.657)**	**<0.001**

^**a**^ **COX regression**

**Fig 1:**
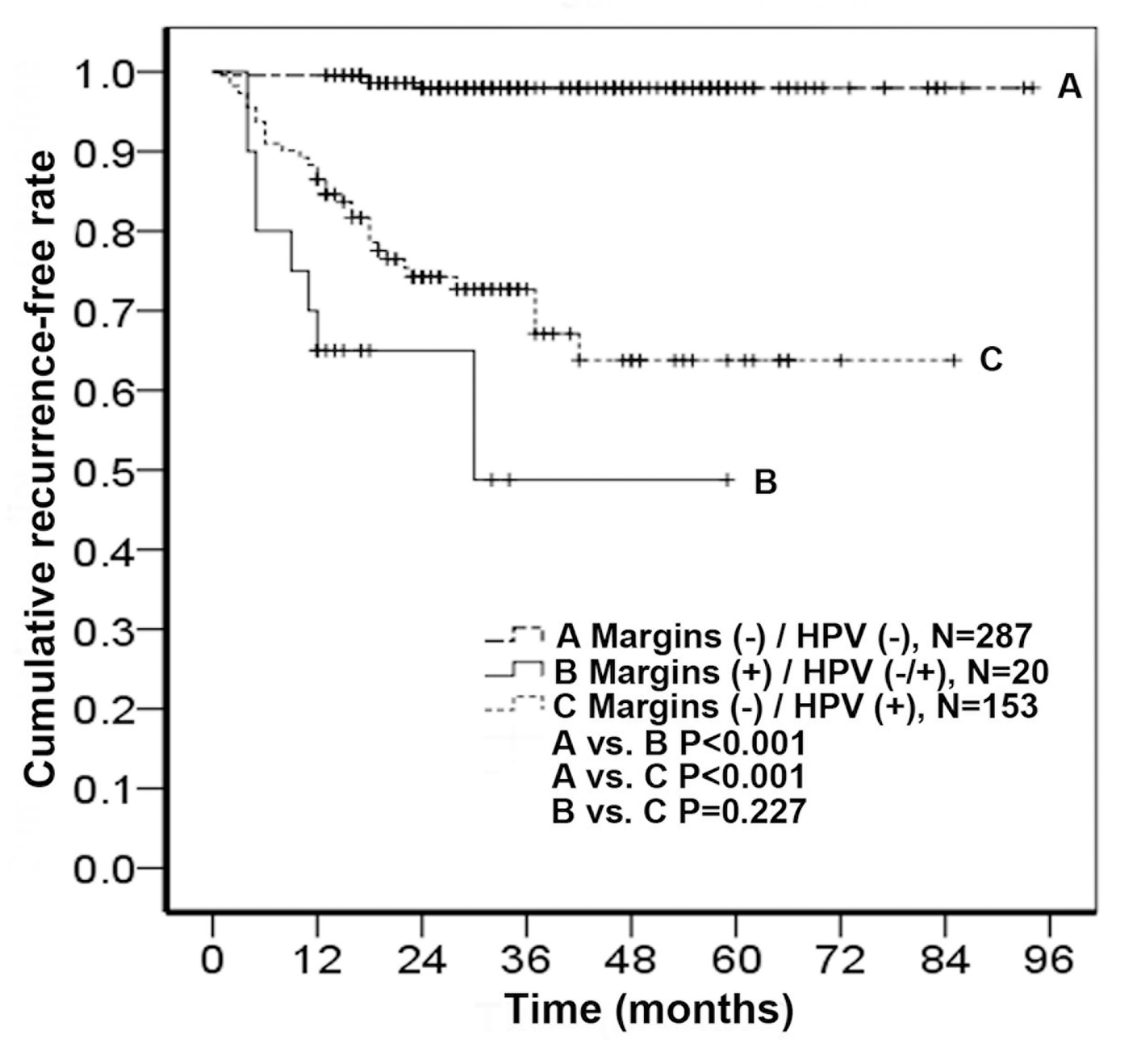
Persistence/recurrence-free rate of women after cervical conization. The patients were divided into three groups according to surgical margin and HPV status.

In order to evaluate the long-term risk of recurrence based on the role of these two predictors, a Kaplan-Meier curve was plotted. We found that women with positive margins had worse outcomes than those with negative margins, and among the patients with negative margins, those with HPV persistence (≥ 6 months) after conization had worse outcomes than those without HPV at 6-months follow-up (Fig 1).

We identified 50 cases with CIN persistence/recurrence and matched these cases with 50 patients without CIN persistence/recurrence (see the matched variables in Table 3). After propensity-score matching, we used SPSS to generate the matched pairs. All the nine matched variables had no significant differences between persistence/recurrence group and no persistence/recurrence. Punch biopsy and colposcopy adequacy were similar between the two groups.

**Table 3:** Characteristics of patients having LEEP/CKC (matched pairs). CKC, cold knife conization; LEEP, loop electrosurgical excision procedure; CIN, cervical intraepithelial neoplasia; HPV, human papilloma virus.

Variable		No recurrence (n = 50)	Recurrence (n = 50)	P value
**Age (median and range)**		**38 (25–54)**	**38 (21–62)**	**0.863** ^**a**^
**Menopause**				**>0.99** ^**b**^
	**Yes**	**3 (6%)**	**3 (6%)**	
	**No**	**47 (94%)**	**47 (94%)**	
**Parity (median and range)**		**1 (0–3)**	**1 (0–3)**	**0.767** ^**a**^
**Conization method**				**>0.99** ^**b**^
	**LEEP**	**19 (38%)**	**19 (38%)**	
	**CKC**	**31 (62%)**	**31 (62%)**	
**Cone depth (mm)**				**>0.99** ^**b**^
	**≤18**	**25 (50%)**	**25 (50%)**	
	**>18**	**25 (50%)**	**25 (50%)**	
**Punch biopsy** ^**d**^				**0.540** ^**b**^
	**CIN1**	**5 (10%)**	**3 (6%)**	
	**CIN2**	**9 (18%)**	**12 (24%)**	
	**CIN3**	**36 (72%)**	**35 (70%)**	
**Colposcopy adequacy** ^**d**^				**0.617** ^**b**^
	**Yes**	**38 (76%)**	**35 (70%)**	
	**No**	**12 (24%)**	**15(30%)**	
**Cone pathology**				**>0.99** ^**c**^
	**Normal**	**3 (6%)**	**3 (6%)**	
	**CIN1**	**5 (10%)**	**6 (12%)**	
	**CIN2**	**9 (18%)**	**9 (18%)**	
	**CIN3**	**33 (66%)**	**32 (64%)**	
**Surgical margins**				**0.524** ^**c**^
	**Negative**	**41 (82%)**	**38 (76%)**	
	**Positive**	**4 (8%)**	**8 (16%)**	
	**Uncertain**	**5 (10%)**	**4 (8%)**	
**Glandular involvement**				**0.668** ^**b**^
	**Yes**	**33 (66%)**	**35 (75%)**	
	**No**	**17 (34%)**	**15 (25%)**	
**HPV persistence(≥ 6 months)**				**>0.99** ^**b**^
	**Yes**	**46 (92%)**	**46 (92%)**	
	**No**	**4 (8%)**	**4 (8%)**	

^**a**^ **Mann-Whitney U test.**

^**b**^ **Pearson Chi-square test.**

^**c**^ **Monte Carlo exact test.**

^**d**^ **not the matched factor.**

The number of quadrants involvement and depth of gland involvement (GI) were studied on H&E sections of the 50 matched pairs. The results are shown in Table 4. According to the number of quadrants involvement, the patients were classified into the following two groups: 1–2 quadrants and 3–4 quadrants. Although the majority of CIN3 lesions involved more quadrants than CIN1-2 lesions (*p* = 0.027), no statistically significant association between more quadrants involvement and persistent/recurrent disease was observed (*p* = 0.398). The depth of GI was less than 3 mm in about 86.8% (59/68) CIN2-3 lesions, while it was 3–5 mm in 11.7% (8/68), and only one CIN3 case had GI of 7.5 mm. No gland involvement was found in 11 CIN1 lesions. The mean depth of CIN2 gland involvement was 0.8 mm (range: 0.2–5 mm), while that of CIN3 was 1 mm (range: 0.2–7.5mm). There was significant difference between the two groups (*p* = 0.039). However, no statistical significance was noted between the persistence/recurrence group and no persistence/recurrence group (*p* = 0.58).

**Table 4:** Number of quadrants involvement and depth of gland involvement in CIN. CIN, cervical intraepithelial neoplasia; QI, quadrants involvement; GI, gland involvement; NA, not applicable.

		No recurrence	Recurrence	P value	CIN1	CIN2	CIN3	P value
**Number of QI**				**0.398** ^**a**^				**0.027** ^**b**^
	**1–2**	**41**	**38**		**11**	**17**	**51**	
	**3–4**	**6**	**9**		**0**	**1**	**14**	
	**Total**	**47**	**47**		**11**	**18**	**65**	
**Depth of GI (mm)**		**1 (0.3–5)**	**1 (0.2–7.5)**	**0.580** ^**c**^	**NA**	**0.8 (0.2–5)**	**1 (0.2–7.5)**	**0.039** ^**c**^
**Cases**		**33**	**35**		**11**	**12**	**56**	

^**a**^ **Pearson Chi-square test.**

^**b**^ **Monte Carlo exact test.**

^**c**^ **Mann-Whitney U test.**

**Fig 2:**
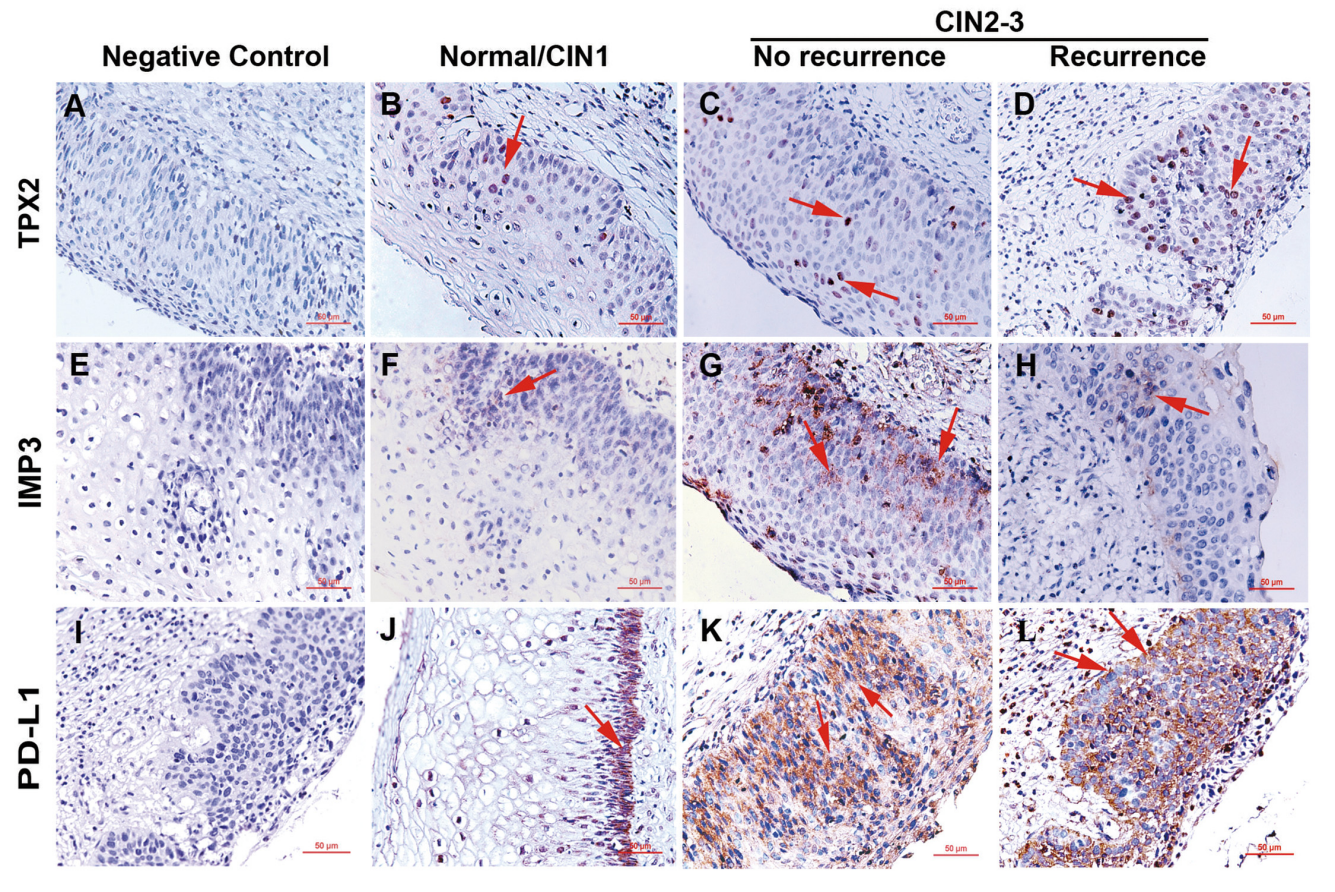
Representive pictures of immunohistochemical staining of TPX2, IMP3, and PD-L1 in cervical tissues. Negative controls used non-immune isotype serum instead of primary antibodies. Arrows indicate the positively stained cells. Scale bars, 50 μm; original magnification, x200.

Then IHC staining results are shown in Table 5. Representative pictures of IMP3, TPX2 and PDL1 staining are shown in Fig 2. For IMP3 staining, cytoplasmic staining in the layer of epithelial cell was scored as positive. The positive rate of IMP3 is 24% (12/50) in no CIN persistence/recurrence group and 22% (11/50) in CIN persistence/recurrence group. IMP3 expression was observed in none of the 6 normal conization specimens, 2 of 11 (18.2%) CIN1, and 21 of 83 (25.3%) high-grade lesion (CIN2 and CIN3) specimens. No significant differences in IMP3 expression were observed between the groups (Table 5).

**Table 5:** Allred score of immunohistochemical staining. CIN, cervical intraepithelial neoplasia; TPX2, xenopus kinesin-like protein 2; IMP3,insulin-like growth factor II messenger RNA (mRNA)-binding protein 3; PD-L1,programmed cell death-1 ligand-1.

Marker		No recurrence (n = 50)	Recurrence (n = 50)	P value	Normal/CIN1 (n = 17)	CIN2-3 (n = 83)	P value
**TPX2**							
	**Intensity score**	**0.7 (0–2.2)**	**1.0 (0–2.2)**	**0.494** ^**a**^	**0.2 (0–1.0)**	**1.0 (0–2.2)**	**0.001** ^**a**^
	**Proportion score**	**1.2 (0–5.0)**	**1.4 (0–4.0)**	**0.639** ^**a**^	**0.4 (0–2.5)**	**1.5 (0–5.0)**	**0.002** ^**a**^
	**Total score**	**2.0 (0–7.2)**	**2.4 (0–6.1)**	**0.624** ^**a**^	**0.6 (0–3.5)**	**2.8 (0–7.2)**	**0.001** ^**a**^
	**Positive staining**	**26 (52%)**	**33 (66%)**	**0.155** ^**b**^	**3 (17.6%)**	**56 (67.5%)**	**<0.001** ^**b**^
**IMP3**							
	**Intensity score**	**0 (0–1.6)**	**0 (0–1.8)**	**0.798** ^**a**^	**0 (0–1.0)**	**0 (0–1.8)**	**0.089** ^**a**^
	**Proportion score**	**0 (0–3.8)**	**0 (0–4.2)**	**0.822** ^**a**^	**0 (0–2.5)**	**0 (0–4.2)**	**0.137** ^**a**^
	**Total score**	**0 (0–5.0)**	**0 (0–6.0)**	**0.865** ^**a**^	**0 (0–3.8)**	**0 (0–6.0)**	**0.124** ^**a**^
	**Positive staining**	**12 (24%)**	**11 (22%)**	**0.812** ^**b**^	**2 (11.8%)**	**21 (25.3%)**	**0.372** ^**b**^
**PD-L1**							
	**Intensity score**	**0.6 (0–2)**	**1 (0–3)**	**0.339** ^**a**^	**0 (0–2)**	**1 (0–3)**	**0.072** ^**a**^
	**Proportion score**	**1.8 (0–5)**	**2.2 (0–5)**	**0.449** ^**a**^	**0 (0–3)**	**2.8 (0–5)**	**0.008** ^**a**^
	**Total score**	**2.5 (0–7)**	**3.2 (0–8)**	**0.380** ^**a**^	**0 (0–5)**	**3.8 (0–8)**	**0.016** ^**a**^
	**Positive staining**	**23 (46%)**	**30 (60%)**	**0.161** ^**b**^	**5 (29.4%)**	**48 (57.8%)**	**0.032** ^**b**^
**TPX2+IMP3Positive staining**		**9 (18%)**	**9 (18%)**	**>0.99** ^**b**^	**1 (5.9%)**	**17 (20.5%)**	**0.280** ^**b**^
**TPX2+PD-L1Positive staining**		**13 (26%)**	**25 (50%)**	**0.013** ^**b**^	**2 (11.8%)**	**36 (43.4%)**	**0.014** ^**b**^
**IMP3+PD-L1Positive staining**		**6 (12%)**	**7 (14%)**	**0.766b**	**0 (0)**	**13 (15.7%)**	**0.176b**
**TPX2+IMP3+PD-L1 Positive staining**		**4 (8%)**	**6 (12%)**	**0.505** ^**b**^	**0 (0)**	**10(12.0%)**	**0.287** ^**b**^

^**a**^ **Mann-Whitney U test.**

^**b**^ **Pearson Chi-square test.**

The expression of TPX2 protein was localized in the nuclei. The positive rates of TPX2 in CIN persistence/recurrence group and no persistence/recurrence group were 66% (33/50) and 52% (26/50), respectively. There was no significant difference between the two groups. However, the intensity and proportion scores and the total Allred score of TPX2 staining as well as the positive rate of TPX2 staining were significantly higher in pathological grade II and III than those in grade I and normal specimens (Table 5).

For PD-L1 staining, expression of PD-L1 on the plasma membrane and/or in the cytoplasm of neoplastic cells was scored as positive. PD-L1 positivity was seen in 60% (30/50) of CIN persistence/recurrence group and 46% (23/50) of no CIN persistence/recurrence group (*p* = 0.161). However, PD-L1 positive rate was significantly higher in CIN2-3 group (57.8%) than that in CIN1/Normal group (29.4%) (*p* = 0.032). There were also significant differences in the proportion score and total Allred score of PD-L1 staining between these two groups (*p* = 0.008 and *p* = 0.016, respective, see Table 5).

Further analysis found that co-expression of TPX2 and PD-L1 was significantly higher in CIN persistence/recurrence group than in no persistence/recurrence group (*p* = 0.013) and there was also significant difference in co-expression of TPX2 and PD-L1 between CIN1/Normal group and CIN2-3 group (*p* = 0.014, see Table 5).

## Discussion

CIN treatment reduces the risk of cervical cancer by 95%, however, the chance of developing cervical cancer in women with CIN is still five times of the general population [5]. It has been suggested that an important reason of persistently increased cancer rate is due to a high rate of default from follow-up [2]. Macgregor JE et al. found that half of those who developed cervical cancer after previous treatment of CIN missed follow-up screening [17]. Identification of the clinicopathologic and/or immunohistochemical predictors of CIN persistence/recurrence may improve follow-up of the high-risk population.

Our findings demonstrate that positive surgical margin is an important predictor for CIN persistence/recurrence after conization, which is consistent with the previous studies [9,11]. In our cohort, CIN persistence/recurrence occurred in 40% of patients with positive surgical margins, which is similar to the previous reports [9,11]. However, only 4.3% patients had positive surgical margins in our cohort, which is lower than the previously reported rates [9,11]. One reason is that some repeat conizations because of positive margin are excluded; another reason is the larger cone size which reduces the possibility of residual lesions. A recent study showed that a cone depth more than 18–20 mm could avoid residual lesions with a 95% probability [18]. In our cohort, approximately 62.7% of the cones had a depth more than 18 mm.

We found CIN persistence/recurrence in 9.5% of the patients with negative surgical margins. In these patients, the CIN persistence/recurrence rate is significantly higher in HPV persistence group than that HPV-negative group (27.2% vs 1.8%, *p* < 0.001). Multivariate analysis showed that HPV persistence for 6 months after conization is another significant risk factor for CIN persistence/recurrence. A systematic literature review found that HPV testing has a high sensitivity in the prediction of treatment failure [3]. In our cohort, approximately 92% CIN persistence/recurrence after conization occurred in women with persistent HPV infections. Approximately 34.8% HPV tests were positive after 6 months of conization, which is higher than the rate (21%) in a previous study [19], but a recent study shows that post-conization persistence 1 year of hrHPV is detected in 21.2% patients[8]. The exact reasons for the discrepancy are not clear, which may be due to multiple factors including personal hygiene, sexual partners, and the HPV test methods.

The number of quadrant involvement was not found to be a predictor for CIN persistence/recurrence in our cohort, which echoes a previous finding by Suttha Hamontri et al [20]. Few studies examined the correlation between the depth of gland involvement and CIN persistence/recurrence. Our data indicated that there was no statistically significant association between them. Nonetheless, we found that the depth of GI was less than 3 mm in most of CIN2-3 lesions and the depth increased from CIN2 to CIN3, which is consistent with a previous study [21]. Whether this finding could be helpful for surgeons to choose surgical scale should be studied further in the future researches.

Further study on Immunohistochemical Predictors of Persistent/Recurrent lesion was carried out after analyzing Clinicopathologic factors. As is known to all, CIN is a premalignant lesion, caused by persistent hrHPV infection. We chose to assess expression of TPX2 as it is related to cellular proliferation, IMP3 as it is involved in cellular invasion, and PD-L1 as it is an immune checkpoint inhibitor that inhibits immune response.

TPX2 is a microtubule-associated protein, and TPX2 overexpression drives carcinogenesis in various malignancies [22]. TPX2 has also been suggested as an early biomarker for malignant transformation of cervical squamous epithelial tissue [14]. IMP3 is an oncofetal protein, which could promote cell adhesion, migration and metastasis in many malignancies [23]. PD-L1 is a cell surface glycoprotein and a member of B7 family of T cell coregulatory molecules, which acts on PD-1 to inhibit T cells [24]. Taku Okazaki et al demonstrated a strong correlation between PD-L1 expression on the tumor cells and a negative prognosis for human cancer patients because PD-1‒PD-L-dependent immune-inhibition is exploited by the tumors to evade the host immune system [15]. We speculate that HPV and CIN neoplastic cells may also activate PD-1-PD-L1 immunosuppression to avoid host immune attacks on the viruses and the abnormal cells. We found that the positive rates of TPX2, IMP3 and PD-L1 expression was 59%, 23% and 53%, respectively. The positive rate of TPX2 expression was similar to the finding of a previous study [22]. The positive rates of IMP3 and PD-L1 were slightly higher than the rates reported in the previous studies [13,25]. It is likely that the main reason is the differences of score systems applied in each study. We did not find any significant differences between CIN persistence/recurrence group and the group without CIN persistence/recurrence, in terms of expression of IMP3, TPX2, and PD-L1. However, we did find that the levels of expression of TPX2 and PD-L1 were significantly higher in the CIN2-3 group than CIN1/normal group, suggesting that these two markers are associated with high grade CIN lesions. In addition, CIN persistence/recurrence group had significantly more cases with co-expression of TPX2 and PD-L1 than the group without CIN persistence/recurrence, suggesting that co-expression of TPX2 and PD-L1 may be a potential predictor for CIN persistence/recurrence.

In summary, the present study found that positive surgical margin, HPV persistence for 6 months after conization, and co-expression of TPX2 and PD-L1 expression are associated with CIN persistence/recurrence after cervical conization. These findings suggest that large cone resection and tests for HPV, TPX2, and PD-L1 may be useful in the clinical treatment and follow-up of patients with CIN.

## Supporting Information

S1 Table Relevant data underlying the findings described in manuscript. (XLSX)
